# Targeting Wnt Signaling in Acute Lymphoblastic Leukemia

**DOI:** 10.3390/cancers17152456

**Published:** 2025-07-24

**Authors:** Samantha Hurwitz, Ki Jun Lee, Tatiana Fourfouris, Irene Choi, Krishan Parikh, Rachel Friedmann, Maiah Zarrabi, Yong-Mi Kim

**Affiliations:** Children’s Hospital Los Angeles, Division of Hematology and Oncology, Department of Pediatrics, Keck School of Medicine, University of Southern California, Los Angeles, CA 90027, USA; shurwitz@chla.usc.edu (S.H.); kjlee@chla.usc.edu (K.J.L.); tfourfouris@chla.usc.edu (T.F.); choiiren@usc.edu (I.C.); ksparikh@usc.edu (K.P.); rfriedmann@chla.usc.edu (R.F.); mzarrabi@chla.usc.edu (M.Z.)

**Keywords:** acute lymphoblastic leukemia, Wnt signaling, Wnt inhibitors

## Abstract

The Wnt signaling pathway plays a critical role in regulating normal hematopoiesis and immune cell development. However, its dysregulation has emerged as a key driver of leukemogenesis. Leukemic stem cells exploit aberrant Wnt signaling to sustain self-renewal, evade apoptosis, and promote unchecked proliferation. In this review, we highlight the dual roles of canonical and non-canonical Wnt pathways in acute leukemia, emphasizing their distinct and overlapping contributions to disease progression. We also evaluate current preclinical and clinical strategies targeting Wnt signaling, identifying both promising advances and persistent obstacles to therapeutic translation. By elucidating the molecular mechanisms underlying Wnt pathway dysregulation in leukemic cells, this review underscores the potential of Wnt-directed therapies as a novel class of interventions to improve outcomes for patients with acute leukemia.

## Simple Summary

Wnt combines “Wingless,” a gene from the fruit fly Drosophila, and “Int-1,” a proto-oncogene from mice. The Wnt signaling pathway helps regulate how blood and immune cells develop normally. However, when this pathway becomes abnormal, it can play a major role in the development of acute leukemia. Leukemia stem cells take advantage of this faulty signaling to keep growing, avoid cell death, and spread uncontrollably. This review explains how different parts of the Wnt pathway contribute to leukemia and how they interact. It also explores experimental and clinical efforts to block Wnt signaling to treat leukemia. While some progress has been made, there are still challenges in turning these discoveries into effective treatments. Understanding exactly how Wnt signaling goes wrong in leukemia cells may open the door to new therapies that could improve survival and outcomes for patients with acute leukemia.

## 1. Introduction to Acute Leukemias

### 1.1. Acute Lymphoblastic Leukemia

Acute lymphoblastic leukemia (ALL) is a severe blood cancer arising from either B-cell lineage (B-ALL) or T-cell lineage (T-ALL) lymphoid precursors, and it comprises a heterogeneous blood malignancy with diverse clinical and molecular features [1]. B-ALL is the most common childhood cancer, accounting for 85% of pediatric ALL [2]. Owing to advances in chemotherapy, immunotherapy, and supportive care, the five-year survival rate of pediatric ALL now exceeds 90% [3]. However, adult patients with ALL face a worse prognosis, with survival rates below 30% [4]. In the past two decades, comprehensive whole-genome analyses have uncovered a wide array of genetic alterations underlying B-ALL, classifying over 20 distinct subtypes linked to treatment outcomes and prognosis [5]. Current subtypes are categorized into favorable or poor risk groups based on chromosomal number—such as high hyperdiploidy or hypodiploidy—and key genetic alterations, including BCR-ABL1, ETV6-RUNX1, IL3-IGH, TCF3-PBX1 fusions, and MLL (KMT2A) rearrangements [6]. T-ALL represents up to 15% of pediatric ALL and 25% of adult ALL cases [7,8], and unlike B-ALL, it is not currently categorized into risk groups based on genetic aberrations [9]. Relapsed T-ALL has a dismal prognosis, with an overall survival rate of less than 25% in children and 10% in adults [10].

### 1.2. Acute Myeloid Leukemia

While the focus of this review is acute lymphoblastic leukemia, it is important to consider the role of Wnt signaling in acute myeloid leukemia (AML), an aggressive hematologic malignancy characterized by abnormal cell proliferation within the myeloid lineage [11]. AML represents only 15% of all acute leukemia diagnoses in children, while in adults it is the most prevalent form of acute leukemia, constituting 80% of cases [12,13,14]. In contrast to ALL, which is often attributed to relatively predictable cytogenetic abnormalities, AML is associated with a highly diverse genetic landscape [15,16,17]. Despite significant improvements in the survival rate of pediatric AML over recent decades, in part due to patient risk stratification and therapy adaptation, the prognosis remains poor, with a survival rate of only 30–40% [16].

## 2. Wnt Signaling

**Figure 1:**
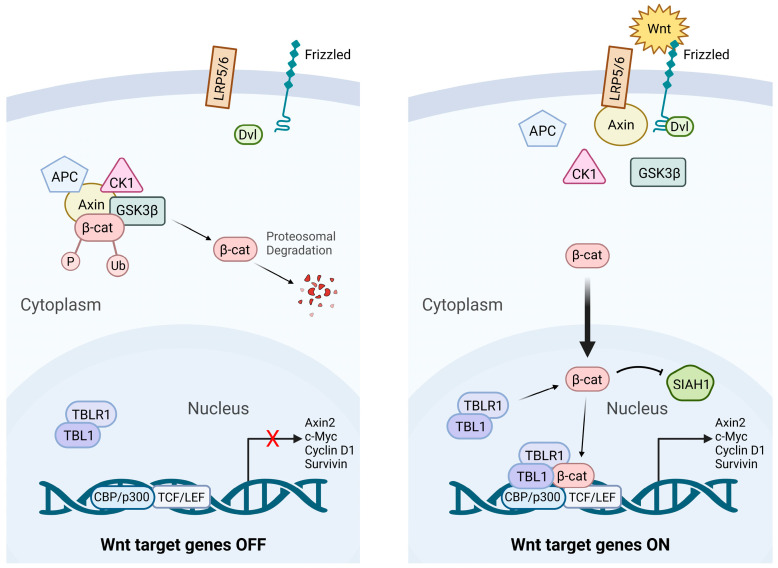
Canonical Wnt/β-Catenin Signaling: “Wnt OFF” vs. “Wnt ON”. Schematic representation of canonical Wnt/β-catenin signaling pathway in inactive (“Wnt OFF”) and active (“Wnt ON”) states. In absence of Wnt ligands (Wnt OFF), β-catenin destruction complex—composed of APC, Axin, CK1α, and GSK3β—phosphorylates β-catenin, leading to ubiquitination and proteasomal degradation, thereby preventing Wnt target gene transcription. When Wnt ligands are present (Wnt ON), they bind to Frizzled receptors and low-density lipoprotein receptor-related protein 5/6 (LRP5/6) co-receptors, activating Disheveled (DVL) and inhibiting the destruction complex. This results in β-catenin stabilization, cytoplasmic accumulation, and nuclear translocation, where it is associated with TCF/LEF transcription factors to activate Wnt target gene expression.

The Wnt/β-catenin signaling pathway is essential for numerous physiological processes, including embryonic development, adult tissue homeostasis, and stem cell maintenance [18]. It achieves this by controlling key cellular functions such as fate specification, differentiation, apoptosis, polarity, and migration [19,20]. Wnt signaling plays a crucial role in the survival and proliferation of lymphocyte progenitors, and it is proposed that the abnormal regulation of this pathway may contribute to the development of lymphoid leukemia [21]. The aberrant activation of the β-catenin, a key component of Wnt signaling, has been implicated in the pathogenesis of various hematological malignancies, including leukemia [22]. Wnt signaling operates through two major branches: the β-catenin-dependent canonical pathway (Figure 1) and the β-catenin-independent non-canonical pathways. While the canonical pathway is well characterized, the non-canonical signaling mechanisms are more diverse and remain comparatively less understood [23].

### 2.1. Canonical Wnt Signaling

The canonical Wnt/β-catenin signaling pathway is an evolutionarily conserved pathway that regulates many cellular functions, such as cell proliferation, migration, apoptosis, self-renewal, and gene expression during embryonic development [24]. β-catenin is the primary mediator of the canonical WNT pathway [25]. In the absence of Wnt ligands, a cytoplasmic destruction complex composed of GSK-3β (Glycogen Synthase Kinase 3β), CK1α (Casein Kinase 1α), Axin (Axis Inhibition Protein), and APC (Adenomatous Polyposis Coli) phosphorylates β-catenin, targeting it for ubiquitination and proteasomal degradation. Upon pathway activation, Wnt ligands bind to Frizzled (FZD) receptors and LRP5/6 co-receptors, leading to the recruitment and activation of Disheveled (Dvl) [26]. This promotes phosphorylation of the LRP5/6 cytoplasmic domains, facilitating Axin binding and disassembly of the destruction complex. Stabilized β-catenin then accumulates and translocates into the nucleus. In the nucleus, β-catenin binds to transducin beta like protein 1 (TBL1) and transducin beta-like protein receptor 1 (TBLR1) complex, which protects its degradation from SIAH1 (Siah E3 Ubiquitin Protein Ligase 1) [27,28]. It can then bind to T-cell factor (TCF)/lymphoid enhancer factor (LEF) [19,29,30]. This transcriptional complex activates target genes that regulate cell proliferation, survival, differentiation, and stem cell maintenance. Overall, Wnt signaling promotes cell division and tissue growth. In the absence of β-catenin, the TCF/LEF complex associates with transducing-like enhancer proteins (TLE/Groucho), which recruit histone deacetylases (HDACs), resulting in transcriptional repression [31]. However, when β-catenin binds to TCF/LEF, it displaces the TLE/Groucho complexes, promoting the recruitment of activators such as CBP/p300 (cAMP-responsive element-binding protein (CREB)-binding protein/EP300/), Pygo, BCL9, and BRG1 to modify the interacting proteins, leading to activation of the Wnt target genes (PDK1, MTC-1, c-Myc, Cyclin D1, Cox-2, Axin2, and survivin) [26,32,33,34,35,36] (Figure 1).

### 2.2. Non-Canonical Wnt Signaling

#### 2.2.1. Wnt–PCP Pathway

The non-canonical Wnt–PCP (planar cell polarity), which is a protein-dependent signaling mechanism that governs the coordinated alignment of cells within the plane of epithelial tissues, regulates cell polarity, movement, and tissue organization [37,38]. It is activated by Wnt ligands, which bind to FZD receptors and activate Dvl proteins [39]. This in turn triggers the activation of small G-protein RhoA (Ras homolog gene-family member A) and Rac1 (Ras-related C3 botulinum toxin substrate 1) [40]. RhoA promotes the activity of Rho-associated kinase (ROCK), while RAC1 enhances the expression of c-Jun N-terminal kinase (JNK), ultimately leading to the activation of downstream target gene expression [37].

#### 2.2.2. Wnt–Ca^2+^ Pathway

The non-canonical Wnt–Ca^2+^ pathway is a signaling cascade activated by Wnt ligands, such as Wnt5a, that bind to FZD receptors. This binding triggers the activation of Dvl proteins, leading to an increase in intracellular calcium (Ca^2+^) levels. The elevated Ca^2+^ activates various downstream signaling effectors, such as protein kinase C (PKC), calcineurin, and calcium/calmodulin-dependent protein kinase II (CaMKII) [41]. These signaling molecules then regulate cellular processes such as gene expression, cytoskeletal rearrangements, and cell migration [42]. In contrast to the canonical Wnt pathway, which is mediated by β-catenin, the Wnt–Ca^2+^ pathway modulates cell behavior and tissue development through calcium-dependent signaling cascades [43,44].

## 3. Wnt Ligand Activation

The Wnt family of secreted glycoproteins plays a crucial role in regulating cell proliferation, differentiation, survival, and oncogenesis. There have been 19 human Wnt proteins discovered thus far [44,45]. These ligands initiate Wnt signaling by binding to Frizzled (FZD) receptors and co-receptors such LRP5/6, leading to the activation of either the canonical (β-catenin-dependent) or non-canonical (β-catenin-independent) pathways [45,46,47,48,49]. Prior to secretion, Wnt proteins undergo palmitoylation by the acyltransferase porcupine (PORCN), a critical step for their receptor binding and functional activity [50].

Wnt1 class proteins are drivers of the canonical pathway, playing a crucial role in promoting cell proliferation, differentiation, maturation, and in establishing proper body-axis specification. Wnt1 class ligands include Wnt1, 2, 3, 3a, 8Aa 8b, 10a, and 10b. Noncanonical Wnt signaling involves Wnt5a-type ligands, such as Wnt4, Wnt5a, Wnt5b, Wnt6, Wnt7a, Wnt7b, and Wnt11 [26,51,52]. The functions of the noncanonical pathway vary, but overall, it is known to play a role in regulating cellular polarization and migration [53,54]. While Wnt ligands have traditionally been grouped into canonical and non-canonical Wnt signaling, accumulating evidence shows that many ligands exhibit context-dependent activity across both pathways. For example, Wnt11 can activate either canonical or non-canonical signaling depending on the receptor composition and cellular environment. Despite this overlap, ligands are grouped here based on their predominant or most studied roles in ALL to help clarity (Table 1).

### 3.1. Canonical Wnt Ligands in ALL

Canonical Wnt signaling is initiated upon Wnt ligand binding to FZD and LRP5/6, resulting in the stabilization and nuclear translocation of β-catenin. This activates TCF/LEF-mediated transcription of target genes.

#### 3.1.1. Wnt1 and Wnt2b

WNT1 and WNT2b are expressed at high levels in acute myeloid leukemia (AML) blasts [55]. WNT2, in particular, is significantly overexpressed in AML, where it promotes leukemogenesis by inducing aberrant gene promoter methylation, thereby enhancing proliferation and reducing apoptosis [40]. This likely occurs through crosstalk with epigenetic regulators such as DNMT3a, TET2, and IDH1/2, which are frequently mutated in AML. These mutations disrupt normal methylation and chromatin remodeling processes, contributing to silencing of tumor suppressor genes and supporting leukemic proliferation [56]. RT-PCR analysis of bone marrow (BM) patient B-ALL samples revealed expression of WNT2b in 33% of cases [57].

#### 3.1.2. Wnt3a

Wnt3A is among the most extensively studied Wnt ligands in ALL. The stimulation of ALL cells with Wnt3a activated canonical Wnt signaling, resulting in increased β-catenin expression and its translocation to the nucleus, leading to increased cell proliferation [57]. Treatment with Wnt3A prompted the accumulation of β-catenin in AML and B-ALL primary cells and cell lines [58,59]. Furthermore, Wnt3A induced the nuclear translocation of β-catenin and activated TCF/LEF1-dependent transcription in the B-ALL cell line NALM6 [59]. In AML and T-ALL cell lines, Wnt3A enhanced clonogenic recovery following suspension culture, indicating a potential role in promoting the self-renewal of leukemic stem or progenitor cells [60]. Overall, Wnt3A supports self-renewal activity in stem/progenitor subpopulations in AML and T-ALL cell lines. Treatment with Wnt3A on AML cell lines led to the activation and nuclear accumulation of β-catenin, as demonstrated by Western blot analysis [58].

#### 3.1.3. Wnt10a and Wnt10b

Wnt10b is a Wnt ligand linked to the regeneration of hematopoietic stem cells. AC133^+^ (a glycosylation-dependent epitope of CD133) cells from AML patients exhibited significantly elevated expression of WNT10B compared to normal AC133^+^ cells, which contain a glycosylation-specific site on CD133 that identifies stem and progenitor cells. This upregulation was further validated by a marked increase in WNT10 protein levels observed in AML patient samples [61,62]. WNT10a expression, in contrast, correlates with favorable prognosis in AML patients treated with chemotherapy, suggesting its potential utility as a prognostic marker [63].

#### 3.1.4. Wnt16b

Studies have shown that Wnt16b plays a role in B-ALL survival. Clinical studies indicate that high expression of Wnt16 is associated with poorer overall survival and higher risk of relapse [64]. Notably, in a pre-B-ALL subtype characterized by at (1;19) translocation, the aberrant E2A-Pbx1 fusion protein activates Wnt16 transcription [65,66]. Moreover, gene expression profiling of leukemic blasts revealed a significant overexpression of Wnt16 in leukemias expressing E2A-Pbx1. When Wnt16b was inhibited, apoptosis was observed [67]. Suppressing E2A-Pbx1 expression results in a significant reduction in WNT-16 mRNA levels, indicating that Wnt16 is a downstream target of E2A-Pbx1 [65].

### 3.2. Non-Canonical Wnt Ligands in ALL

Non-canonical Wnt signaling is β-catenin-independent and primarily includes the planar cell polarity (PCP) and Wnt/Ca^2+^ pathways, which regulate cytoskeletal organization, polarity, and migration [26].

#### 3.2.1. Wnt5a

Wnt5a is an extensively studied non-canonical Wnt ligands that primarily activates the Wnt/Ca^2+^ pathway [26]. In T-ALL, Wnt5a promotes cellular migration and invasion. Treatment of the MOLT4 cell line with Wnt5a significantly increased migratory and invasive capacity as seen through transwell assays [68]. RT-PCR analysis of bone marrow (BM) patient B-ALL samples revealed expression of WNT5a in 42% of cases [65].T-ALL patient samples have also shown high expression of WNT5a, highlighting its role in leukemia progression [69].

#### 3.2.2. Wnt9a

Wnt9a can activate both the canonical and non-canonical Wnt signaling pathways [70]. Wnt9a has functioned as a conserved regulator of hematopoietic stem/progenitor cell (HSPC) development in both zebrafish and humans [71,72]. Wnt5A and Wnt9A transcripts were detected in the stroma-dependent human AML cell line TRL-01 cells, but were absent in hTERT-transduced human bone marrow stroma cell line (HTS) cells. The cell death that was observed in TRL-01 cells following the removal of co-culturing with HTS cells was partially rescued by treatment with Wnt5A or Wnt9A, but not by other Wnt ligands such as Wnt5B [58].

#### 3.2.3. Wnt11

Wnt11 activates both canonical and non-canonical Wnt signaling pathways [73,74]. Pathway enrichment analysis indicated that Wnt11 may contribute to the biological processes of AML by interacting with genes involved in cell morphogenesis during differentiation, blood vessel development, hemostasis, and hematopoietic cell lineage. However, the precise combined mechanism of these factors in AML requires further investigation [63]. Wnt11 is transcriptionally upregulated by the E26 transforming sequence-related gene (ERG) transcription factor, which is commonly overexpressed in AML and T-ALL. ERG-driven WNT11 expression causes morphological changes—cellular elongation and polarization—associated with increased migratory and invasive potential [68]. When studied in patients with AML, high Wnt11 expression negatively impacted survival [62].

#### 3.2.4. Non-Wnt Ligands Modulating Wnt Signaling

In addition to the Wnt activators, several other ligands regulate Wnt signaling by interacting with FZD and/or LRP5/6 receptors [75]. Norrin triggers the canonical Wnt pathway through FZD4 and LRP5, while R-spondin (RSPO) amplifies Wnt signaling via the leucine-rich repeat-containing G protein-coupled receptors (LGR) [76,77,78]. Additionally, the dickkopf-associated protein (DKK) family (DKK1-4) inhibits Wnt signaling by promoting the endocytosis of LRP5/6 receptors. Wnt proteins must also undergo palmitoylation by the acyltransferase porcupine (PORCN) before they can be secreted and bound to these receptors [50].

## 4. Wnt in Acute Lymphoblastic Leukemia

While a majority of research on Wnt signaling in cancer has focused on solid tumors, abnormalities in Wnt pathway activity have also been identified in hematological malignancies [79]. It has been established that abnormal Wnt/β-catenin signaling plays an essential role in AML [80]. However, the involvement of Wnt signaling in ALL remains underexplored. Wnt signaling is essential for preserving the self-renewal and homeostatic balance of HSCs, while also supporting the differentiation and maturation of hematopoietic progenitor cells [51,81]. The activation of canonical Wnt signaling through activated β-catenin has been shown to enhance the self-renewal capacity of hematopoietic stem cells [82]. Deregulation of this signaling network plays a role in the transformation of healthy hematopoietic stem cells (HSCs) into leukemic stem cells (LSCs). LSCs are responsible for sustaining the leukemia cell clone through their capacity for continuous self-renewal, where β-catenin aberrantly activates β-catenin-dependent genes such as survivin, c-Myc, Axin2 or Axin1, TCF1, and LEF1 [25,46,59]. This abnormal and continuous activation of β-catenin is found in both B-ALL and T-ALL and has proven to be essential for maintaining drug resistance [25,37,57,83,84,85,86]. Mutations in either Wnt ligands or β-catenin-dependent genes play a crucial role in the development of ALL. Additionally, many relapsed ALL patients show deleted or decreased expression of known negative Wnt pathway regulators [87].

### 4.1. Wnt Signaling in B-ALL

The significance of the Wnt pathway in normal B-cell development has been demonstrated in two separate models: mice deficient in LEF1 or FZD9 exhibit impaired B-cell development, resulting in a marked reduction in B-cell numbers [22,88,89]. The crucial role that the Wnt pathway plays in B-cell development was first shown in studies where mice lacking LEF1 or FZD9 exhibit disrupted B-cell development and significantly reduced B-cell populations [65]. Pre-B-ALL cells have also been shown to produce Wnt16. Moreover, inhibiting B cell receptor signaling can disrupt Wnt signaling, thereby affecting the survival of pre-B-ALL cell lines [90].

When the Wnt ligand, Wnt-3a, was added to either B-ALL cell lines (NALM6, REH, LK63) or a B-ALL patient sample (0407), β-catenin levels were increased as shown through Western blotting, resulting in significant increases in cell proliferation. The activation of Wnt-3a also inhibited apoptosis in these cells [57]. Moreover, the TCF3-PBX1 (E2A-PBX1) fusion has been linked to increased expression of endogenous WNT16b [67]. Targeted knockdown of WNT16b using siRNA in B-ALL cell lines NALM6 and RCH-ACV was found to induce apoptosis and downregulate survivin (BIRC5), a downstream Wnt gene that promotes ALL cell survival and drug resistance [67,91,92,93,94]. Moreover, it has been shown that Wnt proteins (Wnt2b, Wnt5a, Wnt10b, and Wnt16b) enhance the proliferation of B-ALL cell lines [57] and that Wnt pathway signaling is necessary for supporting the survival of B-ALL LSCs in conjunction with bone marrow stromal cells [95].

Inhibition of the Wnt pathway has also been shown to sensitize B-ALL cells to chemotherapy in vitro and in vivo. B-ALL relapse is correlated with increased survivin expression, giving B-ALL cells a growth advantage [91]. Inhibition of survivin using shRNA in the primary ALL cell LAX7R eradicated minimal residual disease (MRD) in combination with chemotherapy (vincristine, dexamethasone, and l-asparaginase [VDL] for Ph negative ALL or nilotinib, a tyrosine kinase inhibitor, for Ph positive ALL) [91].

### 4.2. Wnt Signaling in T-ALL

The deregulation of the Wnt/β-catenin pathway is common in the development of T-ALL, as the Wnt signaling pathway has been identified as one of the important self-renewal pathways in T-ALL [96,97]. Approximately 80% of pediatric T-ALL patients exhibit elevated levels of β-catenin compared to healthy controls, resulting in abnormal activation of β-catenin-dependent genes such as survivin, c-Myc, TCF1, and LEF [98]. It has previously been seen that T-ALL is dependent on Notch pathway modifications, but due to elevated expression of β-catenin and its cofactor LEF1, as well as the enhanced proliferation seen in T-ALL cells with high LEF1 levels, the Wnt pathway has shown to be a fundamental part of this disease [25,99]. It has more recently been shown that there is a complex interplay between Wnt and Notch signaling pathways contributing to the maintenance of stem-like properties in T-ALL patients [100].

Lymphocyte progenitor cells are influenced by Wnt signals in terms of survival and expansion, potentially mediated by the heightened sensitivity of immature progenitor cells to Wnt proteins [101,102]. Wnt signaling, like Notch signaling, is also essential for the initial stages of T-cell development in the thymus [103]. Thus, it is no surprise that Wnt signaling plays a role in the pathogenesis of various hematological cancers, including CML, CLL, AML, and ALL [104,105]. When β-catenin was inhibited in MOLT-4 T-ALL cell line using an siRNA cocktail comprising four distinct siRNAs to target and inhibit β-catenin, a significant increase in apoptosis was noted in these cells [25]. β-catenin is overexpressed in the majority of T-ALL patients, and populations with high β-catenin levels exhibit strong resistance to treatment [100]. T-ALL subpopulations exhibiting active Wnt signaling are enriched with leukemia initiating cells (LICs), highlighting a link between Wnt pathway activity and the cancer stem cell phenotype in T-ALL [83]. The transcriptional programs dependent on β-catenin in human T-ALL cell lines were explored to investigate the molecular function and clinical relevance of β-catenin in both primary and relapsed T-ALL. They identified a β-catenin-dependent gene signature through ChIP-seq analysis that is specifically associated with treatment failure in T-ALL patients. Importantly, this signature is enriched in genes related to RNA processing. Overall, their findings establish β-catenin as a key regulator of RNA processing in T-ALL and reveal its critical role in the survival of leukemic cells during the recovery phase following chemotherapy after analyzing transcriptomes of T-ALL refractory patients with the worst outcomes [106].

Wnt target genes, including c-Myc, play a central role in cancer progression and are directly engaged in cell growth and the initiation of tumors. A recent study revealed that c-Myc depletion significantly impairs both the growth and proliferation of primary T cells, as well as markedly reduces the expression of metabolism-associated genes [107]. It has also been demonstrated that c-Myc transcription is linked to the development of LICs through the Wnt–β-catenin pathway in T-ALL [108,109,110]. Inhibition of c-Myc, either through small hairpin RNA (shRNA) or pharmacological agents, effectively prevents the onset of leukemia in mice by eradicating the activity of LICs [111]. Thus, c-Myc is a promising target for eradicating LSCs in T-ALL. Additionally, downstream Wnt target gene, survivin and its aberrant expression has been observed in ALL primary cells [86].

### 4.3. Wnt Signaling Aberrations in ALL Pathogenesis

Wnt signaling dysregulation is a hallmark of ALL with aberrant activation promoting leukemogenesis through β-catenin stabilization and nuclear translocation. This leads to transcription of oncogenic targets such as Myc, cyclin D1, Axin2, and Survivin, which drive LIC expansion, G1/S cell cycle progression, and resistance to apoptosis [112,113]. These transcriptional changes are associated with high-risk ALL and poor prognosis, particularly in relapsed or refractory cases [56]. A study by Ng et al. found that over 85% of pediatric T-ALL patients exhibited elevated β-catenin levels and increased expression of Wnt target genes including Axin2, Myc, TCF1, and LEF1, identifying a Wnt-active subgroup with immature immunophenotypes [25].

Epigenetic inactivation of Wnt antagonists including Secreted Frizzled-Related Proteins (SFRP1, SFRP2, SFRP4, SFRP5), Wnt Inhibitory Factor 1 (WIF1), Dickkopf 3 (DKK3), and Hypermethylated in Ductal Carcinoma 1 (HDPR1) through promoter hypermethylation contributes to constitutive pathway activation and correlates with poor prognosis [62].

This hypermethylation phenotype is particularly prevalent in Philadelphia chromosome-positive (Ph^+^) ALL, where at least one Wnt pathway gene was methylated in 66% of patients (n = 75) [114]. Methylation was associated with lower complete remission rates, increased relapse, and dramatically reduced 9-year overall (2% vs. 40%) and disease-free survival (3% vs. 51%). Multivariate analysis confirmed Wnt gene methylation as an independent prognostic factor. WNT5A, a non-canonical Wnt ligand with tumor-suppressive properties, was also frequently silenced by methylation in Ph^+^ ALL, contributing to cyclin D1 upregulation and aggressive disease [114,115].

**Figure 2:**
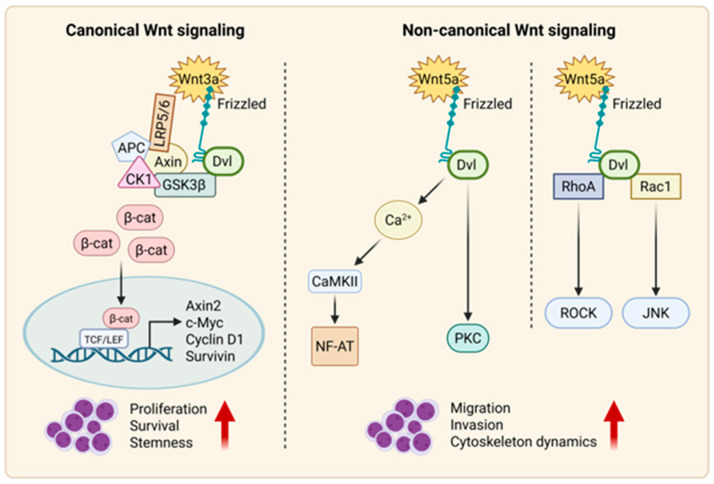
Schematic representation of canonical and non-canonical Wnt signaling pathways and their functional roles in leukemia. Wnt signaling cascade is divided into two major branches: canonical (β-catenin-dependent) and non-canonical (β-catenin-independent) pathways. **Left Panel:** Canonical Wnt signaling: In presence of Wnt ligands (e.g., Wnt3a), binding to FZD and co-receptors LRP5/6 leads to DVL activation and inhibition of β-catenin destruction complex (composed of APC, CK1, GSK3β, and Axin). Stabilized β-catenin translocates into nucleus, where it interacts with TCF/LEF transcription factors to promote transcription of Wnt target genes such as c-Myc, Cyclin D1, Axin2, and Survivin, which drive cell proliferation, survival, and stemness, which are processes often hijacked by leukemic stem cells in ALL. **Right Panel:** Non-canonical Wnt signaling: Wnt5a-class ligands activate β-catenin-independent signaling. Wnt/Ca^2+^ pathway elevates intracellular calcium levels, activating CaMKII and PKC, which in turn stimulate NFAT-mediated transcriptional programs involved in migration and invasion. Planar cell polarity (PCP) pathway involves Dvl-mediated activation of small GTPases RhoA and Rac1, triggering downstream effectors ROCK and JNK. These pathways regulate cytoskeletal dynamics and directional migration which are features linked to leukemic progression.

Moreover, LEF1, a critical TCF/LEF transcription factor, shows subtype-specific alterations. In B-ALL, high LEF1 expression correlates with poor outcomes in adults but favorable prognosis in children. In pediatric T-ALL, LEF1 deletions and mutations often co-occur with NOTCH1 mutations and drive Myc overexpression [62]. Wnt–Notch cross-talk, mediated by regulators such as NRARP (Notch-Regulated Ankyrin Repeat Protein), further modulates leukemic proliferation and Wnt activity [116]. Taken together, these findings underscore the role of Wnt signaling aberrations in ALL pathogenesis, with Wnt target gene expression and methylation status offering both prognostic value and potential therapeutic weaknesses, particularly in certain subtypes such as Ph^+^ ALL. A schematic overview of canonical and non-canonical Wnt signaling pathways and their distinct downstream effects in leukemia is illustrated in Figure 2.

## 5. Leukemia Stem Cells

AML serves as a key model in the development and validation of the cancer stem cell (CSC) theory as the initial isolation of leukemic stem cells (LSC) was in adult AML [117]. Dick et al. discovered that only a subset of leukemic cells bearing the same surface markers as normal adult hematopoietic stem cells (CD34^+^CD38^−^) had the ability to initiate hematopoietic malignancies. These cells were subsequently defined as LICs and are commonly referred to as LSCs or CSCs [118]. However, the CSC theory has not been fully established in ALL. Some of these inconsistencies may stem from the inherent heterogeneity of ALL [119]. Although ALL generally responds well to current therapies and is associated with high long-term survival rates, outcomes decline abruptly in relapsed patients-a phenomenon believed to be driven by the persistence of LSCs [120,121].

### 5.1. Leukemic Stem Cells in B-ALL

In two early studies, Cox et al. [122,123] examined the long-term proliferation of childhood B-ALL cells both in vitro and in vivo. They investigated the expression of several surface markers, including CD34, CD38, CD19, CD133, and CD10, to determine their potential in initiating B-ALL. Their results showed that only a small fraction of B-ALL cells, specifically those with a primitive CD34+/CD10−/CD19−/CD38− phenotype, were able to engraft B-ALL in NOD/SCID mice. Since CD19 is a B-lymphocyte marker and CD10 is associated with lymphocytic differentiation, these findings suggested that like AML, B-ALL originates from a primitive, immature cell rather than a committed B cell [119,122]. Several years later, the team conducted a follow-up study on the expression of the primitive cell marker CD133. They found that only cells with a CD133+/CD19− or CD133+/CD38− phenotype could initiate B-ALL in children, further supporting their earlier conclusions [123,124]. However, several other studies reported contrasting findings, indicating that only the more mature CD19+ B-ALL cells were capable of engrafting as both CD34+/CD38−/CD19+ and the CD34+/CD38+/CD19+ B-ALL cell populations could engraft B-ALL [125,126].

In high-risk childhood ALL patients, including those with MLL gene rearrangement, which is the rearrangment of the histone lysine [K]-MethylTransferase 2A gene (KMT2A) gene on chromosome 11q23, blast cells were identified in three different maturation stages (CD34+CD19−, CD34+CD19+, and CD34−CD19+), all of which were capable of re-establishing and reconstituting the original leukemia phenotype in NOD SCID IL2Rgamma deficient -/-gamma (NSG) mice [120]. These findings highlighted that cells from various maturation stages possess the ability to engraft B-ALL and contain stem cell activity, as both CD19+/CD20− and CD19+/CD20+ cells were shown to successfully engraft B-ALL. However, a study specifically examining MLL-AF4+ infant B-ALL found that only the more mature CD34+CD19+ and CD34−CD19+ populations were capable of engraftment [127]. Similar results were observed in standard-risk patients, such as those with the TEL/AML1 fusion gene, where engraftment was restricted to CD19+ cells [120]. These conflicting findings emphasize the heterogeneity of LSCs in B-ALL and suggest that multiple cytogenetic abnormalities are involved in driving LSCs [128]. In conclusion, evidence shows that B-ALL contains multiple LSC populations with diverse immunophenotypes, making isolation based on surface markers challenging.

Once translocated into the nucleus, β-catenin can interact with co-factors such as CBP or p300. Binding to CBP promotes the expression of genes that support self-renewal, whereas interaction with p300 induces genes that initiate differentiation of CSC/LSC [94]. Among 71 relapsed B-ALL patients and 270 non-relapsed cases, 18.3% of the relapse group exhibited either sequence mutations or deletions in the CBP gene [129,130]. It has been found that CBP/β-catenin antagonists—such as ICG-001—in combination with chemotherapy eradicated drug-resistant CSCs by inducing their differentiation. Notably, ICG-001 effectively eliminates drug-resistant primary B-ALL cells (SFO2, LAX7R) in vitro when used alongside standard therapies, irrespective of CBP mutation status or chromosomal abnormalities. Furthermore, it significantly extends survival in NSG mice engrafted with primary B-ALL [86]. This emphasizes the role the Wnt pathway plays in LSCs in B-ALL.

### 5.2. Leukemic Stem Cells in T-ALL

Identifying LSCs in human T-ALL, like in B-ALL, presents a significant challenge. Recent studies have explored identifying LSCs in pediatric T-ALL using in vitro suspension cultures and in vivo experiments using NSG mice [131]. Efforts have been focused on T-ALL cells expressing CD34 in combination with CD4 and CD7 to assess their potential as LSCs. CD4 is a co-receptor for the T cell receptor, and CD7 marks early T cell differentiation [132,133]. Findings revealed that only cells from the rare CD34+/CD4− and CD34+/CD7− subfractions could engraft T-ALL in mice, suggesting that pediatric T-ALL originates from cells with a primitive immunophenotype, similar to what is observed in AML [131]. However, a study of cortical/mature T-ALL patient samples showed conflicting results. In these patients, LSC activity was confined to the CD34+/CD7+ subpopulation, both in vitro and in vivo, while the primitive CD34+/CD7− cells developed into normal hematopoietic stem cells (HSCs) [133]. Other studies have reported LSC activity in the CD34- population concluding that CD34 is not a universal marker for identifying LSCs in all T-ALL cases [134].

During T-cell differentiation, cells undergo a series of surface marker changes, beginning with a primitive double-negative (CD4−CD8−) stage and sequentially acquiring and losing expression of markers such as CD25 and CD44 [135]. Notably, single-positive populations (CD4+CD8− or CD4−CD8+) also retain the ability to initiate leukemia following transplantation [136].

Another study identified that the CD7+/CD1a− T-ALL cell subset enriched for LSC activity and resistant to glucocorticoids, such as dexamethasone and prednisone, commonly used in T-ALL treatment. Resistance to glucocorticoids is a major factor contributing to treatment failure [134,137]. The CD7+CD1a− subset from primary patient samples exclusively responded to proliferation signals and efficiently initiated leukemia in a xenograft mouse model [134]. This immunophenotype was further established as CD1− cells from high-risk T-ALL showed drug resistance and patients with CD1a− T-ALL had a decreased leukemia-free survival compared to the CD1a+ subtype. Only CD7+CD1a− leukemia cells successfully engrafted in NS122 recipients, while CD7− and CD7+CD1a+ cells showed no engraftment.

Studies have shown that the Wnt/β-catenin pathway is activated in LSCs of both mouse and human T-ALL, contributing to drug resistance The deletion of β-catenin reduced the frequency of LSCs [83]. This implies that targeting the Wnt/β-catenin signaling pathway might be an effective strategy for treating ALL [83]. However, the involvement of the Wnt/β-catenin network in T-ALL pathogenesis is highly complex, and the exact mechanisms driving ALL development are still not fully understood. β-Catenin is known to influence LIC involvement in T-ALL, but its role in sustaining LSCs is not well understood [138]. Elevated levels of β-catenin have been noted in LICs across both human and mouse models of T-ALL, underscoring the essential role of β-catenin signaling in the maintenance and advancement of T-ALL. Through coimmunoprecipitation followed by liquid chromatography–mass spectrometry a noncanonical functional interaction was revealed between β-catenin and the Forkhead box O3 (FOXO3) transcription factor. FOXO3 positively regulates LIC-related genes, including cyclin-dependent kinase 4, a key regulator of the cell cycle and tumor maintenance, which is mainly expressed in cell subsets of MRD [138].

A study demonstrated that conditional deletion of a single allele of the β-catenin gene significantly reduces the frequency and delays the onset of T-ALL driven by Pten loss, suggesting that β-catenin pathway activation may play a role in the initiation or expansion of the LSC population [139]. To validate this, Weng et al. introduced a real-time fluorescent Wnt reporter construct into primary mouse T-cell leukemias through lentiviral transduction and discovered that LICs are concentrated in a small tumor subpopulation that exhibits active Wnt signaling. In a complimentary experiment, genetic inactivation of the Wnt pathway by blocking β-catenin using a lentivirus encoding dominant negative TCF (dnTCF) lacking the N-terminal β-catenin binding domain (dnTCF) into human T-ALL cell lines (HPBALL and RPMI 8402), substantially reduced the frequency of LICs and led to survival prolongation in xenografted T-ALL in mice [83].

Overall, distinctive markers that reliably identify LSCs in B-ALL are still lacking compared to myeloid leukemias. The stem cell biology of lymphoid leukemias remains less understood mainly due to the complexity and variability within leukemias, even when examining each subtype individually.

## 6. Targeting Wnt Signaling in B-ALL and T-ALL

**Figure 3:**
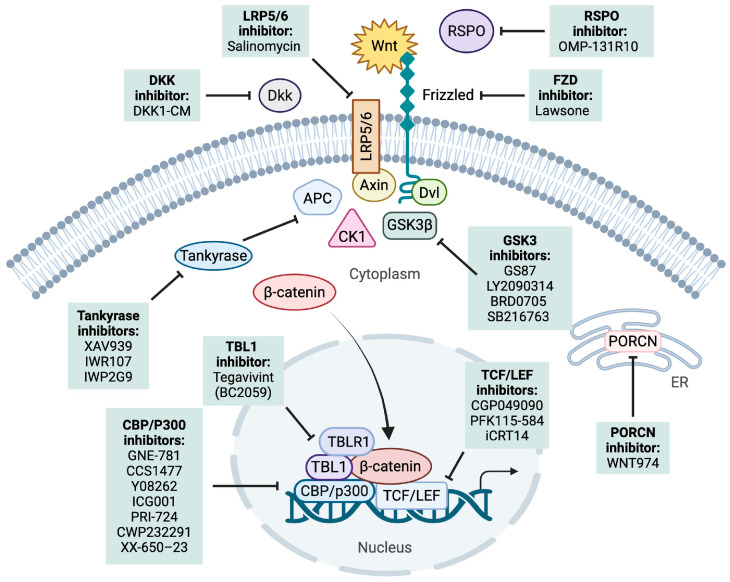
Therapeutic targeting of the Wnt/β-Catenin pathway in leukemia. Graphical overview of pharmacological interventions targeting components of the canonical Wnt/β-catenin signaling pathway in acute leukemia. Therapeutic strategies include inhibition of Wnt ligand–Frizzled (FZD) receptor interactions, modulation of the β-catenin destruction complex, and blockade of β-catenin–TCF/LEF-mediated transcription. Several of these agents are under active preclinical or clinical investigation. APC: Adenomatous polyposis coli gene; AXIN: Axis inhibition protein; CBP: CREB binding protein; CK1: Casein kinase 1; DKK: Dickkopf-1; DVL: Disheveled; FZD: Frizzled; GSK3β: Glycogen synthase kinase 3β; LRP: Low density lipoprotein receptor-related protein; P300: E1A binding protein 300; PORCN: Porcupine; RSPO: R-spondin; TCF/LEF: T-cell factor/lymphoid enhancer factor.

There have been various approaches explored to target acute leukemias [37,140]. Targeting the Wnt/β-catenin pathway has been shown to play a role in minimizing the self-renewal capacity of drug-resistant LSCs [91,107,140,141,142,143]. Here we discuss the different approaches to targeting the Wnt pathway preclinically and clinically for acute leukemias. This includes targeting either upstream of the pathway, promoting β-catenin degradation, or inhibiting the nuclear TCF/LEF complex (Figure 3) (Table 2).

### 6.1. Upstream Effector Targeting

#### 6.1.1. PORCN

Wnt974 is an inhibitor of the PORCN enzyme, which is a membrane-associated protein belonging to the O-acyltransferase family that modifies Wnt proteins through palmitoylation. WNT974 treatment in K562 AML cell line lowered Wnt pathway targets such as ROR2, LRP6 and GSK3B protein and Axin2 protein expression. Treatment also reduced the self-renewal capacity of primary AML patient cells, without significantly affecting apoptosis or cellular quiescence shown through a Colony Forming Unit (CFU) assay. Lastly, using a Mll^PTD/WT^/Flt3^ITD/WT^ double knock-in CN-AML mouse model, an aggressive AML model, the research showed a significant decrease in murine Myc expression in spleen cells utilized for the primary transplantations after treatment with Wnt974. However, Wnt974 treatment on its own did not affect LSC activity in vivo [144].

#### 6.1.2. DKK1

DKK1, a Wnt signaling antagonist [161], inhibits the Wnt signaling pathway through two potential mechanisms; DKK1 not only prevents the formation of the Fz-LRP6 complex but also interacts with the LRP/Kremen co-receptor complex, promoting the internalization of Wnt proteins and thereby reducing the intensity of Wnt signaling [24]. When DKK1 was applied to treat BALL-1/vincristine resistant (VCR) cells, it inhibited the Wnt/β-catenin pathway and resulted in an elevated level of chemoresistance in these cells. The activation of the Wnt/β-catenin pathway can be competitively inhibited by the secreted Wnt antagonist, DKK1.

#### 6.1.3. LRP5/6

Salinomycin inhibits Wnt signaling through the coreceptor LRP6 degradation. In CLL cells with persistent Wnt activation, nanomolar concentrations of salinomycin significantly downregulated the expression of Wnt target genes, including LRP6. Salinomycin treatment in primary cells resulted in the loss of leukemia repopulation ability after transplantation, as indicated by prolonged recipient survival compared to controls. AML and MLL cell lines, along with primary cells and patient samples, were all sensitive to submicromolar concentrations of salinomycin. This treatment effectively hindered the repopulation of leukemia in primary cells post-transplantation, leading to enhanced survival of the recipient mice [145]. Notably, elevated LRP5/6 expression is linked to ALL pathogenesis and may serve as a marker of disease severity and risk [146].

#### 6.1.4. RSPO-LGR4

Salik et al. identified RSPOs and their receptor leucine-rich repeat-containing G-protein coupled receptor 4 (LGR4)—a positive modulator of the canonical Wnt signaling pathway—as key regulators that upregulate critical self-renewal genes and are essential for LSC self-renewal in certain AML subsets. RSPO is a secreted protein that binds to LGR4 within the Wnt pathway. Salik et al. found that clinical-grade anti-RSPO3 antibody, OMP-131R10/rosmantuzumab, disrupted self-renewal and promoted differentiation in AML patient-derived xenografts, while leaving normal hematopoietic stem cells unaffected [162]. 

#### 6.1.5. FZD

Multidrug resistance (MDR) poses a significant challenge in the treatment of cancer. Hamdoun et al. developed novel compounds to combat MDR leukemia. They tested lawsone derivatives against the drug-sensitive CCRF-CEM T-ALL cells. Compound (**1**) (3-hydroxy-1,4-dioxo-N-phenyl-naphthalene-2-carboxamide), which binds to Frizzled-7 and Frizzled-8, exhibited the highest activity, effectively reducing the protein expression of β-catenin, c-Myc, Pgp, and Frizzled-7 in a dose-dependent manner [163].

#### 6.1.6. GSK-3

GSK-3 is a serine/threonine kinase with two isoforms: GSK3α and GSK3β [147]. It plays a multifaceted role in cancer, acting as both a tumor suppressor and promoter depending on cellular context. Inactivation of GSK3 has been associated with decreased overall survival in AML [148], though in other cell types, inactivation may be beneficial. Inhibition of its activity causes a loss of phosphorylation sites, abnormal stabilization of β-catenin, and a subsequent increase in TCF-dependent gene expression [164]. While GSK3α is less studied in the context of Wnt signaling, recent work has shown that selective inhibition of GSK3α (e.g., BRD0705) can suppress leukemia initiation without promoting β-catenin stabilization, offering a potentially safer therapeutic avenue [146]. Among its functions, GSK3β is a key regulator of the Wnt signaling pathway [165]. In the absence of Wnt ligands, GSK3β forms a destruction complex with APC and Axin that phosphorylates β-catenin, targeting it for proteasomal degradation. Inhibition of GSK3β disrupts this process, leading to β-catenin stabilization, nuclear translocation, and TCF/LEF-dependent gene expression. In leukemia cells, this activation can have pro- or anti-survival consequences, and has been shown to induce apoptosis by suppressing the expression of survival genes such as Bcl2 and survivin [166].

A GSK3 inhibitor, GS87, was developed through efforts to enhance GSK3 inhibition for AML differentiation. Not only did GS87 induce AML differentiation, but kinase profiling also showed that GS87 specifically targeted GSK3 with minimal activity against other kinases. GS87 demonstrated strong efficacy in a mouse AML model and had minimal impact on normal bone marrow cells. GS87 represents a novel GSK3 inhibitor with significant therapeutic potential as a differentiation agent for non-promyelocytic AML [149].

In preclinical murine models of mixed-lineage leukemia (MLL), inhibiting GSK-3 showed preliminary antitumor efficacy, strengthening the notion that GSK-3 inhibitors could serve as a potential novel treatment option for hematological malignancies [167]. In non-clinical studies, the GSK-3 inhibitor, LY2090314, inhibited cell viability and induced apoptosis in vitro in AML cell lines, MLL-translocated leukemia, erythroleukemia, CML, T-ALL (Eli Lilly and Company, Indianapolis, IN). There was also an open-label, Phase 2 study that evaluated the safety of LY2090314 in patients with AML. The data indicated that single-agent LY2090314 demonstrated an acceptable safety profile but offered limited clinical benefit in AML patients at the investigated dose and frequency [150] (NCT01214603).

A GSK3α-selective compound, BRD0705, also showed to inhibit leukemia initiation and extend survival in an AML mouse model. BRD0705 blocked kinase activity without stabilizing β-catenin, thereby minimizing potential neoplastic risks. It induced myeloid differentiation and disrupted colony formation in AML cell lines (MOLM13, TF-1, U937, MV4–11, HL-60 and NB4) and patient cells, while showing no effects on normal hematopoietic cells and shows therapeutic efficacy in vivo across multiple xenograft and syngeneic mouse models of AML driven by the oncogene *MLL-AF9* [168].

Inhibiting GSK3β kinase causes significant proliferation defects in various leukemia cell types, leading to apoptosis [167,169]. To assess the impact of GSK3β activity in leukemia cells, Zhou et al. exposed T-ALL JURKAT cells, CML K562 cells, and myeloma RPMI-8226 cells to the GSK3β kinase inhibitors LiCl and SB216763. This inactivation caused growth inhibition of the leukemia cells while also inhibiting the activation of the survival genes Bcl2 and survivin by c-Myc and LEF-1 [166].

### 6.2. Promoting β-Catenin Degradation

#### Tankyrase

XAV939 is a small molecule inhibitor of tankyrase, a multifunctional poly (ADP-ribose) polymerase that stabilizes the axin complex in the Wnt/β-catenin signaling pathway. Preclinical studies showed XAV939 effectively inhibited Wnt/β-catenin signaling and induced apoptosis in leukemic cells. It also diminished β-catenin expression and reduced chemoresistance both in vitro and in vivo in B-ALL cell lines (REH, RS4;11, and SEMK2) [95]. XAV-939 was also able to reduce the malignant phenotypes in T-ALL cells overexpressing the trans-activation response DNA binding protein (TARDBP) [151]. TARDBP is part of the heterogeneous nuclear ribonucleoprotein family and plays a role in RNA processing, including transcription, transport, splicing, and stability [152,153,159]. Microarray analysis of pediatric T-ALL bone marrow samples revealed an upregulation of TARDBP. Mai et al. tested XAV-939 to determine if TARDBP is involved in T-ALL phenotype through β-catenin. They discovered that Inhibition of β-catenin with XAV-939 effectively blocked cell proliferation and cycle progression while promoting apoptosis in TARDBP-overexpressing T-ALL cells (JURKAT and MOLT4) [151].

Previous studies have demonstrated that the transcriptional activator β-catenin plays a role in promoting MLL-AF9-driven leukemogenesis, while the mechanism is not well understood. Zhang et al. identified Wnt acyltransferase PORCN and Tankyrase enzymes as essential regulators of a WNT-SIX1 signaling axis that enhances cell growth in MLL-AF9-expressing leukemic cells [170]. The Tankyrase inhibitor IWR107 and IWP2G9 reduced the progression of MLL-AF9 fusion AML by interfering with Wnt–β-catenin–SIX1 signaling in THP1, MV4;11, HL60 and U937 AML cells. IWR107 was chemically modified based on the structure of IWR1 [154,170]. IWR107 and IWP2G9 effectively inhibit Wnt signaling in cultured cells by reducing both cytoplasmic and nuclear biochemical markers as each compound either suppresses recombinant Tankyrase activity or blocks Wnt protein fatty acylation, as revealed by a click chemistry-based assay.

### 6.3. Inhibiting β-Catenin–TCF Interaction

#### 6.3.1. TCF

In the Wnt pathway, β-catenin acts as a crucial co-activator, binding to TCF/LEF transcription factors in the nucleus to drive the expression of target genes involved in cell proliferation, survival, and differentiation, thus playing a pivotal role in the regulation of cellular fate decisions [29]. Pre-treatment of a β-catenin/TCF inhibitor, iCRT14, on purified blasts from relapsed leukemia patients followed by prednisolone exposure, successfully restored chemosensitivity in these cells. Furthermore, treating B and T-ALL cell lines (REH, NALM6, UOCB1 and MOLT-4 cells) with iCRT14 caused a notable downregulation of Wnt target genes. When combined with standard chemotherapeutic drugs (etoposide, prednisolone, and doxorubicin), this approach resulted in a synergistic reduction in cell viability and a significant increase in apoptotic cell death [85].

#### 6.3.2. LEF-1

CGP049090 and PFK115-584 are both LEF/β-catenin inhibitors, suppressing transcription of Wnt target genes. When treated by both compounds, AML cell lines Kasumi-1 and HL-60 showed significant killing of AML cell lines along with primary AML blasts, while PBMCs were not significantly affected. There was also a significant reduction in the expression of β-catenin/LEF1 target genes, including c-Myc, cyclin D1, and survivin, demonstrating the specificity of these inhibitors [155]. More specifically, PKF115-584, which disrupts the β-catenin and LEF1 complex, prevented and even partially reversed leukemogenesis by inducing apoptosis and decreasing proliferation in human T-ALL cells (RPMI8402, HPB-ALL, JURKAT, CCRF-CEM) [108] and inhibited survival of T-ALL leukemic cells in vivo. Interestingly, high expression of LEF-1 is recognized as a positive prognostic factor in childhood ALL [171], though the utility of inhibitors against this molecule in these patients is yet to be examined.

#### 6.3.3. CBP/P300

Pharmacological inhibition of CBP has demonstrated preclinical efficacy across various AML subtypes by triggering cell cycle arrest and promoting apoptosis [172]. Although several CBP inhibitors have been identified, few have advanced into clinical trials for AML. Consequently, there remains a strong need for the development of new, potent, and selective CBP inhibitors with diverse structural scaffolds. GNE-781, an inhibitor of the CBP and P300 bromodomain, demonstrated strong antiproliferative effects against hematologic cancer cell lines, exhibited favorable pharmacokinetic characteristics, and showed encouraging antitumor activity in an AML MOLM-16 xenograft model [173,174]. However, preclinical safety evaluations revealed that GNE-781 caused adverse effects on hematopoietic, gastrointestinal, and reproductive tissues [174].

CCS1477, a first-in-class oral CBP and P300 bromodomain inhibitor, is currently undergoing early-phase clinical trials (NCT04068597) for the treatment of hematologic malignancies and advanced solid tumors. In an AML xenograft model, CCS1477 achieved complete tumor suppression during a 21-day treatment period, with no tumor regrowth observed for up to 14 days post-treatment. Despite these advances, the number of CBP bromodomain inhibitors progressing to clinical trials for AML remains limited [156].

The CBP bromodomain inhibitor, Y08262, has newly been tested for treating AML as part of the continued efforts to discover novel CBP bromodomain inhibitors with distinct and diversified chemical scaffolds. This study introduces a novel lead compound that supports further validation of the CBP bromodomain as a therapeutic target for AML drug development The new inhibitor also exhibits strong inhibitory effects across AML cell lines [157].

ICG-001 is a small molecule inhibitor that specifically targets prevents the interaction between β-catenin and CBP [158]. Inhibiting the CBP/β-catenin interaction has driven LSCs towards differentiation to return cells back to a normal HSC state. ICG-001 is in early-phase clinical trials for hematological malignancies and for solid tumors (NCT01606579). Several studies have emphasized that Wnt/β-catenin signaling regulates the early stages of normal T-cell development, while its dysregulation can contribute to the malignant transformation of T-cell progenitors [139,175]. Through testing ICG-001 on T-ALL cell lines (CEM-S, JURKAT, HPB-ALL, MOLT-4, RPMI-8402), Evangelisti et al. demonstrated that simultaneous targeting of the Wnt/β-catenin pathway using ICG-001, and the PI3K/Akt/mTOR axis with ZSTK-474, a PI3K inhibitor, effectively reduced the proliferation, survival, and clonogenic activity of T-ALL cells [158]. ICG-001 and ZSTK-474 exhibited cytotoxic effects, and their combination led to a marked increase in apoptotic cells. This apoptosis induction was linked to the downregulation of the Wnt/β-catenin and PI3K/Akt/mTOR pathways [158]. While it is established that the PI3K/Akt/mTOR and Wnt/β-catenin pathways are implicated in leukemogenesis, the effects of concurrently inhibiting both signaling pathways have not been explored in T-ALL. ICG-001 can trigger differentiation in drug-resistant primary pre-B-ALL cells, enhancing their sensitivity to both targeted and conventional chemotherapy, thus overcoming drug resistance. ICG-001 treatment resulted in the eradication of drug-resistant primary B-ALL when combined with conventional therapy in vitro and significantly extends the survival of NSG mice engrafted with primary ALL (LAX7R, LAX3, TXL3) [86].

Protein tyrosine phosphatase of regenerating liver 3 (PRL-3) is overexpressed in AML patients, highlighting its potential as a therapeutic target. The dual PI3K/mTOR inhibitor VS-5584 and the WNT inhibitor ICG-001 were used in combination to exploit synthetic lethal interactions between the mTOR/AKT and WNT/β-catenin pathways. This drug combination significantly reduced leukemic burden and improved survival in mice transplanted with PRL-3-high AML cells but had no effect on mice with PRL-3-low AML cells [160].

Jiang et al. found that PRI-724 (a prodrug of C-82 and analog of ICG-001), a second-generation selective antagonist of CBP/β-catenin, promotes apoptosis, inhibits cell growth, and downregulates β-catenin target genes in AML cell lines (MOLM13 and OCI-AML3) as well as in stem/progenitor cells. When treating AML cell lines in combination with PRI-724, and chemotherapy sorafenib, the combination synergistically induced apoptosis AML cell lines, AML blasts, and FLT3-mutated CD34+CD38− AML stem/progenitor cells. This combination also reduced nuclear β-catenin, c-Myc, and other proteins involved in Wnt/β-catenin signaling. Lastly, in a MOLM13-GFP/Luc cell xenograft in NSG murine model, each agent extended the survival of mice compared to the control; however, the combination treatment of PRI-724 with sorafenib extended survival the most [176].

CWP232291 (CWP291) is a small-molecule compound that interferes with the association between CBP and β-catenin [177]. It inhibits the Wnt signaling pathway through triggering endoplasmic reticulum (ER) stress, which activates caspases that decrease β-catenin levels. The unfolded protein response (UPR), a signaling cascade triggered by ER stress, is recognized as a vital mechanism in the survival of cancer cells [178]. Recent studies highlight the critical role of the UPR in the development and progression of acute leukemias [179]. In preclinical studies, CWP291 showed considerable antitumor activity in both cell cultures and animal models, including bone marrow engraftment models of AML and various other cell lines [180]. In a Phase 1 study, CWP232291 was found to be safe and showed single-agent efficacy in AML patients, with plans for upcoming combination trials [181] (NCT01398462). Patients with ALL have not been included in trials thus far.

XX-650–23 is another inhibitor of CBP that has been shown to trigger apoptosis and cell cycle arrest in AML cells (KG-1, HL-60, MOLM-13, MV-4-11, and U937), while also extending survival in vivo in mice engrafted with patient AML cells (HL-60) [182]. XX-650–23 also demonstrated promising preclinical effectiveness when combined with the chemotherapy, dasatinib in pre-BCR+ ALL [183], overall uncovering new factors that influence sensitivity to targeted therapies in human pre-BCR+ ALL.

#### 6.3.4. TBL1

Transducin-like protein 1 (TBL1) is a key co-activator in the Wnt/β-catenin signaling pathway. It interacts with the TCF/LEF transcription factors and β-catenin, facilitating the transcriptional activation of Wnt target genes [28]. BC2059 (Tegavivint) is a small molecule inhibitor of Wnt/β-catenin pathway specifically binding to TBL1, disrupting the binding of TBL1 to β-catenin, leading to the degradation of β-catenin. It has been previously shown that Interfering with the TBL1 and β-catenin interaction eliminates β-catenin’s pro-proliferative and tumor-promoting signaling [27,28]. BC2059 has shown efficacy in AML, ALL, as well as other cancer types (NCT04874480, NCT04851119, NCT04780568, NCT05755087) [184]. Fiskus et al. discovered that BC2059 treatment interferes with the β-catenin–TBL1 interaction, resulting in proteasome-mediated degradation of β-catenin in AML cells leading to apoptosis in primary AML blast progenitor cells (BPCs). BC2059 and/or panobinostat markedly enhances survival in NSG mice with OCI-AML3 xenografts and in NSG mice engrafted with primary AML blasts [185]. There is also a phase 1 trial for relapsed or Refractory AML that is active, but not recruiting (NCT04874480).

## 7. Conclusions

In conclusion, targeting key components of the Wnt signaling pathways holds great potential for advancing therapeutic strategies in acute leukemia. Disrupting aberrant Wnt signaling—whether through inhibition of specific ligands, receptors, or downstream effectors—has shown the ability to modulate leukemic cell proliferation and apoptosis in various ways. While preclinical studies have demonstrated encouraging results, further investigation is necessary to refine these strategies for clinical application. Some approaches have already exhibited promising therapeutic effects in clinical trials, suggesting that Wnt signaling inhibitors could offer a valuable addition to current leukemia treatments. Continued research into the complex roles of Wnt signaling will be crucial for optimizing these therapies, avoiding toxicities, and improving patient outcomes.

## Figures and Tables

**Table 1:** Wnt ligands and their roles in acute lymphoblastic leukemia.

Wnt Ligand	Signaling Type	Role in ALL
Wnt1	Canonical	Drives β-catenin activation; involved in proliferation and differentiation
Wnt2b	Canonical	Linked to leukemogenesis and methylation-mediated gene regulation in B-ALL
Wnt3a	Canonical	Promotes proliferation, β-catenin activation, and self-renewal
Wnt10a	Canonical	Prognostic marker in AML; not well-studied in ALL
Wnt10b	Canonical	Linked to HSC regeneration and leukemia stemness
Wnt16b	Canonical	Promotes survival in pre-B-ALL with E2A-Pbx1 fusion
Wnt5a	Non-Canonical	Enhances migration and invasion in T-ALL
Wnt9a	Canonical/Non-Canonical	Supports leukemic cell survival via stromal interaction
Wnt11	Canonical/Non-Canonical	Induces morphological changes and invasion

**Table 2:** Preclinical and clinical inhibitors targeting the Wnt/β-Catenin pathway in acute leukemia.

Targets		Compound	Leukemia Type	Clinical Trial (Number)	PMID	
**Targeting Upstream Effectors**	PORCN	WNT974	AML	Preclinical	[144]	
	DKK1	DKK1-conditioned medium	B-ALL	Preclinical	[24]	
	LRP5/6	Salinomycin	AML	Preclinical	[145]	
	RSPO	OMP-131R10 (rosmantuzumab)	AML	Preclinical	[162]	
	Frizzled	Lawsone	T-ALL	Preclinical	[163]	
	GSK3	GS87	AML	Preclinical	[149]	
		LY2090314	AML	NCT01214603, Phase II	[167]	
		BRD0705	T-ALL	Preclinical	[168]	
		SB216763	T-ALL		[166]	
**Promoting** **β-** **catenin Degradation**	Tankyrase	XAV939	B-ALL, T-ALL	Preclinical	[95,151]	
		IWR107	AML		[170]	
		IWP2G9	AML		[170]	
**Inhibiting β-catenin–** **T-cell factor (TCF) Interaction**	TCF	iCRT14	B-ALL, T-ALL	Preclinical	[85]	
	LEF1	CGP049090	AML	Preclinical	[155]	
					[155]	
		PFK115-584	AML			
			T-ALL		[108]	
	CBP/P300	GNE-781	AML	Preclinical	[173]	
		CCS1477		NCT04068597 Phase 1/2a	[156]	
	CBP	ICG-001	T-ALL	Preclinical	[158]	
			B-ALL		[86]	
			AML		[160]	
		PRI-724 (C-82 pro-drug)	AML	NCT01606579 Phase I/II	[176]	
		CWP232291	AML	NCT01398462 Phase I	[180]	
		XX-650–23	AML, B-ALL	Preclinical	[182,183]	
		Y08262	AML	Preclinical	[157]	
	TBL1	BC2059	AML	NCT04874480 Phase I	[185]	
